# Supplementary material for: XRE transcription factors conserved in Caulobacter and φCbK modulate adhesin development and phage production
Source: PLoS Genet. 2023 Nov 16;19(11):e1011048. doi: 10.1371/journal.pgen.1011048 (PMC10688885; doi:10.1371/journal.pgen.1011048)
Supplement: Attachment — Submitted filename: Reviews.docx [file pgen.1011048.s012.docx]

We thank the reviewers for their time, and for their thoughtful feedback on our manuscript. Based on reviewer comments, we have conducted new experiments and have revised the manuscript accordingly.

We feel that this manuscript is much improved, and hope that it is now suitable for publication in PLoS Genetics.

Best regards,

Sean Crosson

**Reviewer #1 –**

Summary:

This manuscript from McLaughlin et al. reports on the family of XRE type-transcription factors in Caulobacter crescentus and in related phages. This family has undergone an astounding expansion in different lineages of Caulobacter and in related Alphaproteobacteria. The study does a very nice genomic comparison and then drills down into the functions for a few of these in C. crescentus, focusing most on the phiCbK phage encoded regulator. The overlapping specificity and pervasiveness of the XRE family is intriguing, and does invoke interesting ideas such as the concept of ecoparalogs and regulatory feedbacks in bacterial and phage systems.

Major Comments:

This is an excellent study. The experimentation and comparative bioinformatics is compelling and well performed, and the writing is clear and insightful. I have no major qualms with the quality of the data, or the information reported.

I would suggest that the authors spend a bit more time explaining and perhaps diagramming the structure of the XRE transcription factors. They are treated as regulatory actors rather than as complex molecules in the paper, and a bit more info on how they function would strengthen the manuscript. They are short proteins comprised predominantly of a HTH motif. CI and Cro have been beaten to death and so by comparison, the other XREs may work similarly? A little more info on projected mechanism of action would be helpful.

Response: Thank you for the positive feedback. We have added a new discussion section near line 400 that describes mechanisms known to regulate XRE-family TFs in different model systems, including CI from phage λ, SinR from *B*. *subtilis*, and the XRE homologs in Alphaproteobacteria.

Minor Comments:

1. L124. It would be worth mentioning that holdfast production in M2X is usually quite low due to the inhibitory activity of hfiA among other things, compared with rich medium such as PYE. Otherwise, readers may question the strikingly low low values in Fig. 4B.
Response: We agree and have added a sentence to the text (L119) to address this point.

2. L244-45. Were these all the same positions for the fusions? N-terminal or C-terminal with T18/T25.

Response: We have revised the text near line 340 to state that these were C-terminal fusions.

**Reviewer #2 –**

Summary:

This manuscript by McLaughlin, et al explores members of the XRE family of transcription factors identified through a pangenome analysis of Caulobacter. Using ChIP-seq and RNA seq, they show that XRE proteins encoded in Caulobacter and in the phiCbK phage can control transcription of overlapping sets of genes including holdfast regulators and gene transfer agents. Additional RNAseq experiments support roles for the phage encoded XRE being expressed early stages of infection, suggesting an important role for this factor in phage infectivity. These XRE proteins seem to be quite redundant and ectopic expression of any of the paralogs in a ∆xre background can increase phage burst size and suppress gafYZ/hfiA to varying degrees. The XRE proteins interact with themselves and each other based on bacterial two-hybrid results and the authors discuss how this heteromeric interaction may lead to complex regulation. The authors conclude that XRE genes play multiple roles, including adhesion regulation and host-phage dynamics during infection. Overall, the work is extensive, resulting in a wealth of new binding site data and transcriptional results, the experiments are well done, and the interpretations are clearly supported by the data. Some lingering questions remain as described below, the first three of which in this reviewers' opinion could benefit from experiments (but not critically), while the fourth could be addressed relatively easily.

Major Comments:

1. It was ultimately unclear what the link was between XRE function in the host (holdfast production, GTA regulation, etc) and why the phage encoded TgrL would be important to express early for the infection. The authors addressed this discordance in the discussion, where they mention that phage infection does not lead to change in expression of the holdfast regulators (line 328 and table S3). It is appreciated that this would be challenging to fully address, but the absence of experimental connection between the molecular pathways controlled by XREs and the role of TgrL in phage infections slightly reduces the overall enthusiasm. Of course, if the authors show that ∆tgrL phage are not deficient in infectivity, then that would also clarify this point in that it would show this gene is not important for that process.
Response: We agree with these points raised by the reviewer. Our data showing that *tgrL* is a strong early gene suggests it has a role in subverting host defenses and/or preparing the cell for replication of the phage (as is seen in early regulators from other phage). This manuscript defines regulatory targets of TgrL and host XREs and reports infection phenotypes, but the mechanism(s) by which XRE regulated transcription at particular promoters ultimately impacts phage production remain undefined. This is a line of research that we hope to pursue going forward, and we have enhanced our discussion to include possible mechanisms that may underpin the infection phenotype (based on the seq results). We are also excited about deleting phage genes, like *tgrL*, but have not yet been successful in implementing approaches to knock out (or knock down) genes from phiCbK. TgrL is competent to regulate *hfiA*, but the impact of TgrL binding at this promoter in the context of infection also remains to be determined. We have revised writing around line 479 to emphasize that *hfiA* is controlled by an ensemble of distinct transcription factors, and is also strongly nutritionally regulated. Additional experiments are needed to test how phiCbK TgrL and changes in the physicochemical state of the environment combinatorially intersect to impact regulation of the *hfiA* promoter.

2. Similarly, what role do host XREs play in the phage burst size effects? Based on the RNAseq, the XREs themselves are not induced upon phage infection either, so why would deletion of these factors reduce burst size by 50% (Figure 9). Could this be a more indirect effect? For example, are there changes in growth, development, or cell surface that make Δ*xre* strains less susceptible? Is there a change in the timing of lysis either seen in population measurements or microscopically? Again, it is appreciated that this may be challenging to address experimentally, but at a minimum, a deeper description of this aspect of the host XREs in phage virulence would be good in the discussion.
Response: To begin to address this question experimentally, we performed RNA-seq on wild type (WT) *C. crescentus* and used this data set to define transcriptional differences between WT & quadruple Δ*xre* (the data are in revised Table S4). Expression of several toxin and nuclease genes was elevated in the Δ*xre* background. Toxins and nucleases have been implicated in host defense in other systems. In the discussion, near line 568, we further discuss that one possible explanation for the reduced burst size in the Δ*xre* background is that this strain has increased expression of potential phage defense systems, which may prime it to defend against phage infection.

3. The authors mention that *cdxB* was a hit in the Christen study looking at genes important for phiCbK infection. Were any of the other XREs also implicated? If not, is cdxB the major driver of phenotypes for the Δ*xre* strain? This should be brought up in the text or tested.
Response: We agree that this is an important point. To address this question, we assayed phage burst size in the Δ*cdxB* mutant and observed a reduction in the burst size compared to the wild type, similar to the Δ*xre* background. Figure 9 has been updated with the new data, and writing in the results and discussion has been updated to describe this important result.

4. The authors make a compelling argument that XRE proteins can form heteromers based on their bacterial two hybrid results. As described in the discussion, this additional multimerization could lead to a rich control of regulation by different combinations of XREs. However, almost because it was referred to so much, it would be useful to have some experimental data to back up this claim. For example, the authors note that TgrL XRE interacts with itself as well as it does with CdxA and CdxB. Does ectopic expression of TgrL in the presence of either of these XREs change the regulation of hfiA or *gafYZ*? This could be addressed with either co-expression experiments, or in genetic backgrounds like the Δ*xre* strain and adding back one or both of the CdxA/B encoding genes. This could be done for other combinations of XRE as well (or instead of the TgrL work) but some experimental evidence for the importance of XRE heteromeric activity would strengthen the impact of this aspect of the work substantially.

Response: We agree with the reviewer that this is an interesting line of inquiry. We performed RNA-seq overexpressing *tgrL* in a wild type (WT) background (i.e. containing the host XRE proteins) and in a Δ*xre* background. While we observed differences in expression of some genes in the WT versus Δ*xre* strain, we hesitate to interpret these results too deeply (with regard to heteromerization) as the data are confounded by other factors. Specifically, expression of the host XRE factors changes upon *tgrL* overexpression (e.g. *rtrA* is downregulated 2.5-fold; *cdxA* and *cdxB* are upregulated 3-fold). A thorough investigation of the role of heteromeric interactions will require rigorous biochemical approaches that we are working to develop for these closely related proteins but do not yet have in place. We have revised the manuscript in places to make it clear that the functional importance of heteromerization observed in the two hybrid system (if any) remains to be determined in *Caulobacter*.

5. The authors are commended for laying out a wonderfully clear depiction of ideas and results. The supplemental tables are extensive but allow afficionados to see the details without losing the importance of the bigger picture. This attention to all aspects of this work are appreciated.

Response: We greatly appreciate reviewer 2’s kind comments. We hope that investigators find these datasets useful in the future.

Minor comments:

1. Line 168 should be Figure 6C I think.

Response: We have corrected line 168 to say Figure 6C
